# Polypyrrole/reduced graphene oxide composites coated zinc anode with dendrite suppression feature for boosting performances of zinc ion battery

**DOI:** 10.1038/s41598-022-12657-9

**Published:** 2022-05-23

**Authors:** Sonti Khamsanga, Hiroshi Uyama, Weerapong Nuanwat, Prasit Pattananuwat

**Affiliations:** 1 Department of Materials Science, Faculty of Science, Chulalongkorn University, Bangkok, 10330 Thailand; 2 Department of Applied Chemistry, Graduate School of Engineering, Suita, Osaka 565-0871 Japan; 3 Center of Excellence On Petrochemical and Materials Technology, Chulalongkorn University, Bangkok, 10330 Thailand; 4 Research Unit of Advanced Materials for Energy Storage, Chulalongkorn University, Bangkok, 10330 Thailand

**Keywords:** Batteries, Batteries

## Abstract

Metallic zinc (Zn) anode has been received a great promise for aqueous rechargeable zinc-ion batteries (ZIBs) due to its intrinsic safety, low cost, and high volumetric capacity. However, the dendrite formation regarding the surface corrosion is the critical problems to achieve the high performance and the long lifespans of ZIBs. Here, we purpose the facile cyclic voltammetry deposition of polypyrrole/reduced graphene oxide (PPy/rGO) composites coated onto Zn 3D surface as Zn anode for ZIBs. As results, the deposited PPy/rGO layer demonstrates the homogeneous distribution covering onto Zn surface, effectively suppressing the formation of dendrite. Additionally, a symmetric cell of the PPy/rGO coated Zn remarkably enhances an electrochemical cycling with a low voltage hysteresis for zinc plating/stripping, which is superior to the pristine Zn cell. In addition, the deposited layer of PPy/rGO on Zn effectively improves the reactivity of electrochemically active surface area and the intrinsic electronic configurations, participating in extraction/intercalation of Zn^2+^ ions and leading to enhance ZIBs performance. The coin cell battery of Zn-PPy/rGO//MnO_2_ can deliver a high initial discharge capacity of 325 mAh/g at 0.5A/g with a good cycling stability up to 50% capacity retention after 300 cycles. Thus, these achieved results of Zn-PPy/rGO//MnO_2_ battery with dendrite-free feature effectively enhance the life-performance of ZIBs and open the way of the designed coating composite materials to suppress dendrite issues.

## Introduction

Recently, Metal ion (Li^+^, Mg^2+^, Zn^2+^, etc.) batteries (MIBs) play an important role in renewable energy of clean technology as electrochemical energy storage^1–4^. Lithium-ion batteries (LIBs) are widely desirable for the large-scale energy storage because of their high energy density, high operating voltage and excellent cycling stability^5^. However, the lack of lithium resources and the safety concerns cannot be passed over in LIBs^6,7^. To solve these limitations, the other metal ion batteries have paid attention such as sodium-ion batteries, aluminum-ion batteries, potassium-ion batteries and so on^4,6,8^. Among of them, zinc-ion batteries (ZIBs) are considered to be the promising alternatives due to low cost, environmental-friendly issue and high theorical capacity (820 mA h g^−1^)^3,9–12^. Currently, the optimization of electrode materials, electrolytes and other components have been studied to improve the electrochemical performances of ZIBs. Particularly, zinc anode, which is a key material affecting to energy density and cycling performance, has always been received attention from many researchers^13–19^.

The metallic zinc foil is common anode material for ZIBs because of its abundant resources, ability to recycle and high productivity^19–21^. Despite these advantages, there are still some serious problems that need to be addressed in electrochemical reaction between zinc anode and electrolyte interface. One critical issue related to hydrogen evolution reaction (HER) is took place during charged/discharged process with an aqueous electrolyte system, causing to consume the surface anode and to increase the interface impedance^18,22–24^. In addition, the non-uniform growth of Zn dendrite during battery cycling can affect to the decrease battery performances and the internal short-circuit^25,26^.

Various strategies have been explored to dispatch these issues, such as surface modification of zinc foils^27^, incorporation of zinc powder with conductive materials^14^, coating surface of Zn with protection layer^22,23^ and so on^25,28–30^. Among these strategies, the zinc electrodeposition on the host conductive current collectors (Ni foams, ZnO, Zn/Al alloys, etc.) can be noticeable due to the uniform zinc surface and the good electrostatic attraction. However, the corrosion of zinc anode is still appeared because of the occurring of a side reaction of HER. Thus, more achievable approaches of surface engineering for improving Zn surface have been demonstrated to protect the corrosion from HER. More importantly, the coverable material with high electrochemical surface activity is also required for Zn surface modification. Principally, several reports demonstrated the use of metallic oxides, polymers, and carbon-based materials as coating material on Zn surface. Bhoyate and coworkers reported that the formation layer of MoS_2_ in 2D vertical structure on Zn surface by electrochemical deposition can provide the uniform plating/stripping of ZIBs^31^. Zhao and coworkers proposed the use of polyamide coated Zn surface as an anode to create the nucleation barrier, leading to extend the lifetime of Zn anode^25^. Wang and coworkers investigated the use of carbon black coated Zn as anode, resulting in terminating of the dendritic growth and side reactions^28^. However, those of metallic, polymer and carbon materials are low conductivity and the cover coating on zinc surface is difficult to control. In addition, the conductive polymers (CP) such as polyaniline and polypyrrole are principally considered as conductive materials, which can form tightly as nanoparticles on surface. The in-situ Zn coating process with conductive materials revealed the benefiting process to achieve the very low internal resistance at zinc/conductive materials interface. Yong and coworkers reported the using polyaniline coated onto zinc to inhibit corrosion reactions, affecting to decrease a self-discharge behavior and to reduce the HER in batteries^32^. Therefore, the combination of the excellent conductivity materials, the good affinity to zinc-ion, and the large specific surface area materials are required to promote the performances of zinc anode^22,28^. In particular, the in-situ composite coating technique with conductive materials is attractive concept in that two types of materials have been simultaneously grown on same substrate.

Herein, the in-situ coating of polypyrrole (PPy) and reduced graphene oxide (rGO) using cyclic voltammetry technique has been carried out to produce the layer composites on zinc anode for improving the performance of aqueous ZIBs. PPy and rGO are typical conductive polymer and graphitic carbon, respectively. The better wetting ability of PPy to aqueous electrolyte, compared with inorganic protective layer, is expected to promote the zinc-ion diffusion, resulting in improving the surface interfacial interaction and leading to the well uniform of the zinc growth^22,33^. Meanwhile, an atomically thin sheets of carbon atoms of rGO can benefit in view of zinc-ion distribution and protective layer, which can control Zn dendrite formation and corrosion from side reaction of HER^34–36^. Dendrite suppression has been continuously investigated by the method of covering with carbon materials but up to our knowledge there is no report about the use of PPy/rGO composites as the protective layer on Zn anode surface to inhibit the formation of dendrite for ZIBs.

Therefore, in this study, the as-prepared Zn anode sample with the deposited layer of PPy/rGO at different ratios was examined to determine their physical characterization. Subsequently, the electrochemical properties and performances of ZIBs of the different Zn-modified anodes were investigated and discussed. It is challenging that the combination of conductive materials could lead to enhance the electrochemical performance of ZIBs.

## Experimental

### Chemical and materials

ZnSO_4_.7H_2_O (99.0% purity, KEMAUS) was purchased from Elago Enterprises Pty. Ltd. Pyrrole (99.0% purity, TCI), purchased from Tokyo Chemical Industry Co., Ltd., was distilled under reduced pressure and stored in the dark before use. Other the reagents used were of analytical grade and were used as received without any further purification. Nickel foam (0.5 mm thick, 100 PPI) was purchased from Qijing Trading Co., Ltd. Whatman glass microfiber filter was purchased from GE Healthcare Life Sciences. Carbon fiber paper (180 µm thick, 62 g/cm^3^) was purchased from Fuel Cell Store.

### Preparation of graphene oxide (GO) aqueous dispersion

GO was synthesized by the modified Hummers’ method as described elsewhere^37,38^. Briefly, 3.0 g of graphite and 1.5 g of NaNO_3_ in 69 ml of concentrated H_2_SO_4_ were mixed at 4 °C in the ice bath. Then, the first portion of 9.0 g KMnO_4_ was added as oxidizing agent and kept stirring at 35 °C for 7 h. Subsequently, the second portion of KMnO_4_ (9.0 g) was added and employed at 50 °C for 7 h. The reaction mixture was poured into the large portion of ice water and 6 ml of H_2_O_2_. The filtrated products were several washed with 10%w/v HCl, purified by dialysis and dry at 60 °C for 24 h to obtain GO powder. For exfoliated GO aqueous solution, GO powder was dispersed in DI water (1.0 mg /ml) with the aid of ultrasonic bath (50 Hz) for 6 h and centrifuged at 3,000 rpm for 15 min to remove the unexfoliated GO.

### Preparation of Zn-PPy and Zn-PPy/rGO anode

**Figure 1:**
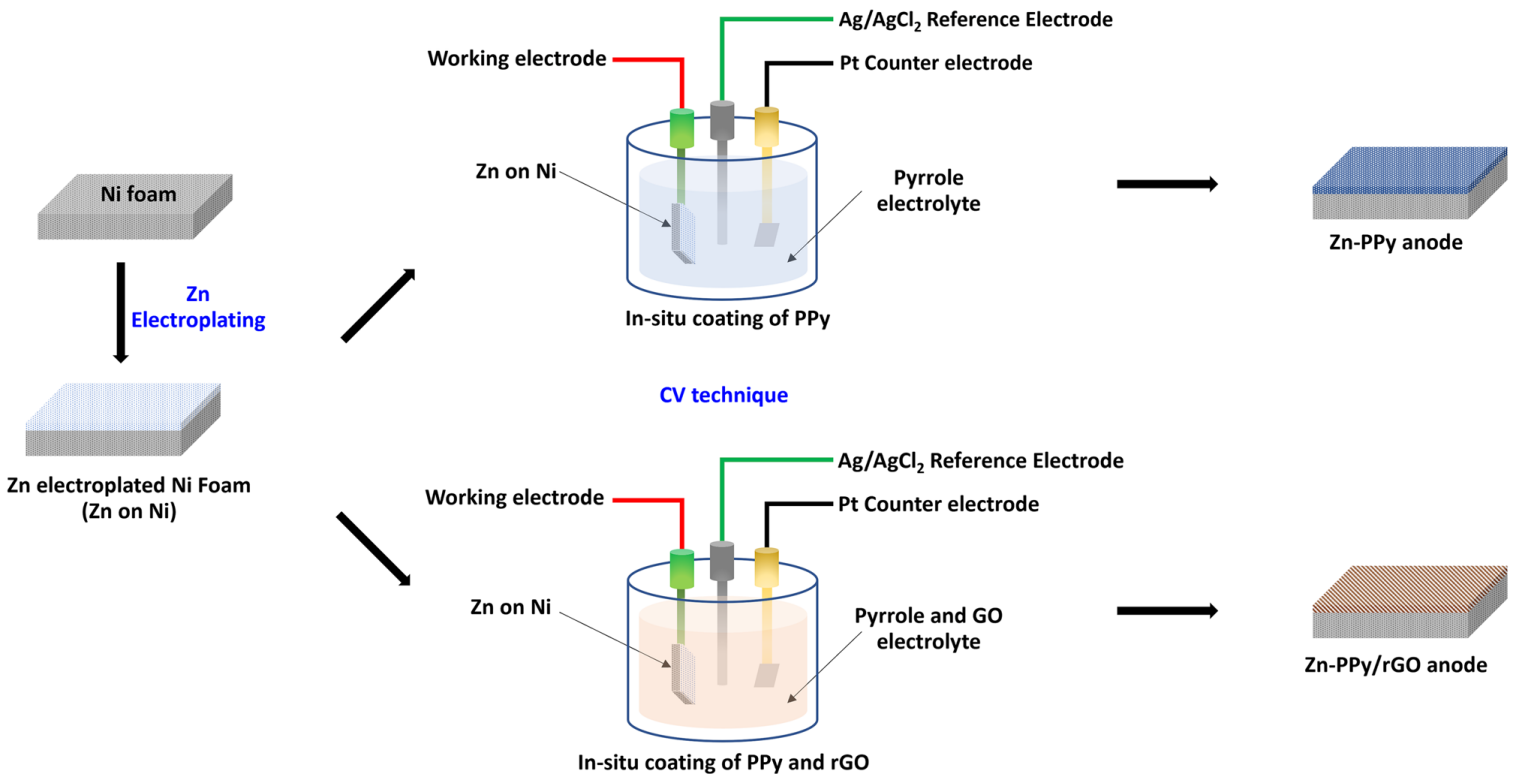
Schematic of the preparation process of Zn-PPy and Zn-PPy/rGO anode.

PPy/rGO coated zinc (Zn-PPy/rGO) onto nickel foam was prepared with two-steps method, including of electroplating and cyclic voltammetry technique. Firstly, the current density of 65 mA/cm^2^ was applied onto the parallel electrode system consisting of nickel foam as cathode and zinc plate as anode with 0.6 M zinc sulfate aqueous electrolyte for 10 min by using Potenstat/galvanostat (Metrohm Autolab, PGSTAT30). The deposited zinc onto nickel surface was further dried for 12 h in vacuum oven. Secondary, the three-electrode system, configurating with Zn coated nickel foam as working electrode, Ag/AgCl as reference electrode and platinum as counter electrode, was employed with 50 ml of electrolyte at 5 mV/sec in the potential window between − 1.0 to 1.0 V for 5 cycles. 50 ml of electrolyte, consisting of 0.05 mg/ml GO aqueous dispersion, 2.0 M of pyrrole and 1.0 mg/ml of sodium dodecyl sulfate (SDS), was adjusted pH to 2 by 0.1 M H_2_SO_4_ before used. Then, the as-prepared anode was washed and dried in vacuum at 60 °C for 12 h. The different concentrations of the dispersed GO in electrolyte were also prepared at 0, 0.001, 0.05, and 0.1 mg/ml as aforementioned method and denote as Zn-PPy, Zn-PPy/rGO001, Zn-PPy/rGO005 and Zn-PPy/rGO01, respectively. The schematic design for Zn-PPy and Zn-PPy/rGO anode preparation are illustrated as shown in Fig. 1.

### Preparation of δ-MnO_2_ cathode

δ-MnO_2_ was synthesized by hydrothermal method as described elsewhere^3^. Briefly, 0.948 g of KMnO_4_ was dissolved in 35 ml of DI water. Then, the 0.169 g of MnSO_4_⋅H_2_O was added to KMnO_4_ solution, and continuously stirring for 30 min. Subsequently, the mixture was transferred into a Teflon autoclave and kept at 160 °C for 12 h in an oil bath. The product was collected and washed with DI water and ethanol several times. Then, it was dried at 60 °C for 12 h. The δ-MnO_2_ cathode was fabricated by pasting the mixtures of 85%wt. of δ-MnO_2_, 10%wt. of conductive carbon and 5%wt. of polytetrafluoroethylene (PTFE) in N-methyl pyrrolidinone (NMP). The mixture slurry was pasted on carbon fiber paper current collector, dried in vacuum oven at 60 °C for 3 h and pressed by compression roller. The mass loading was about 3 mg/cm^2^.

### Characterizations and electrochemical measurements

X-ray Diffraction (XRD, Bruker AXS Model D8 Discover) with Cu Kα radiation at a scanning range of 10 to 90° was carried out to determine the crystallinity structure of Zn, Zn-PPy and Zn-PPy/rGO anode. Field Emission Scanning Electron Microscope (FESEM, FEI-Quanta250FEG) configurating with EDS mode was used to observe the surface morphology and to analysis the surface element composition. X-ray photoelectron spectroscopy (Scanning XPS Microprobe, ULVAC-PHI PHI 5000 Versa ProbeII) was used to investigate the chemical states of samples.

The coin-cell assembly was constructed with δ-MnO_2_ as the cathode, Zn, Zn-PPy and Zn-PPy/rGO as the anode with Whatman glass microfibers as the separator using 2.0 M ZnSO_4_ aqueous solution as electrolyte. The cathode, anode and separator were cut into a disk and assembled into coin cell using a CR2032 standard cell. Cyclic Voltammetry (CV) was performed by Potenstat/galvanostat (Metrohm Autolab, PGSTAT204) at a scan rate of 1.0 mV/s in the voltage range 0.8 to 2.0 V and − 1.2 to 1.2 V vs Zn^2+^/Zn for the coin and symmetrical cell, respectively. The symmetrical cells of Zn, Zn-PPy and Zn-PPy/rGO anode was tested to investigate the cycling performance of plating/stripping at a current density of 0.5 mA/cm^2^ with a cycle time of 15 min. To evaluate the corrosion of Zn, Zn-PPy and Zn-PPy/rGO anode, potentiodynamic polarization (PDP) technique, as illustrated by Tafel extrapolation, was applied to conventional three-electrode, configurating with the as-prepared anode as working electrode, platinum plate as the counter electrode and Ag/AgCl as the reference electrode in electrolyte of 2.0 M ZnSO_4_. The response current densities of the as-prepared anodes were monitored at the scan rate of 0.5 mV/s with the potential range of − 1.0 to − 0.9 V versus with its open circuit voltage (OCV). A battery testing system (BTS-5V10mA, Neware) was used to study the galvanostatic charge/discharge (GCD), rate capability and cycling stability of the coin cell. Furthermore, Nyquist plot was carried out using an Electrochemical Impedance Spectroscopy (EIS) technique of Chemical Impedance Analyzer (IM3590, HIOKI) using an amplitude of 20 mV in a frequency range of 0.1–100,000 Hz.

## Results and discussion

In this study, the formation of the deposited PPy/rGO layer on zinc surface is simultaneously synthesized with two mechanisms during electrodeposition including of electrochemical polymerization of pyrrole and reduction of GO. Accordingly, the polymerization of pyrrole is carried out with 6 step mechanisms: pyrrole oxidation, dimer formation, oxidation, coupling, re-aromatization, and chain propagation^39^. While GO is also simultaneously converted to the rGO by oxygen reduction^40^.

**Figure 2:**
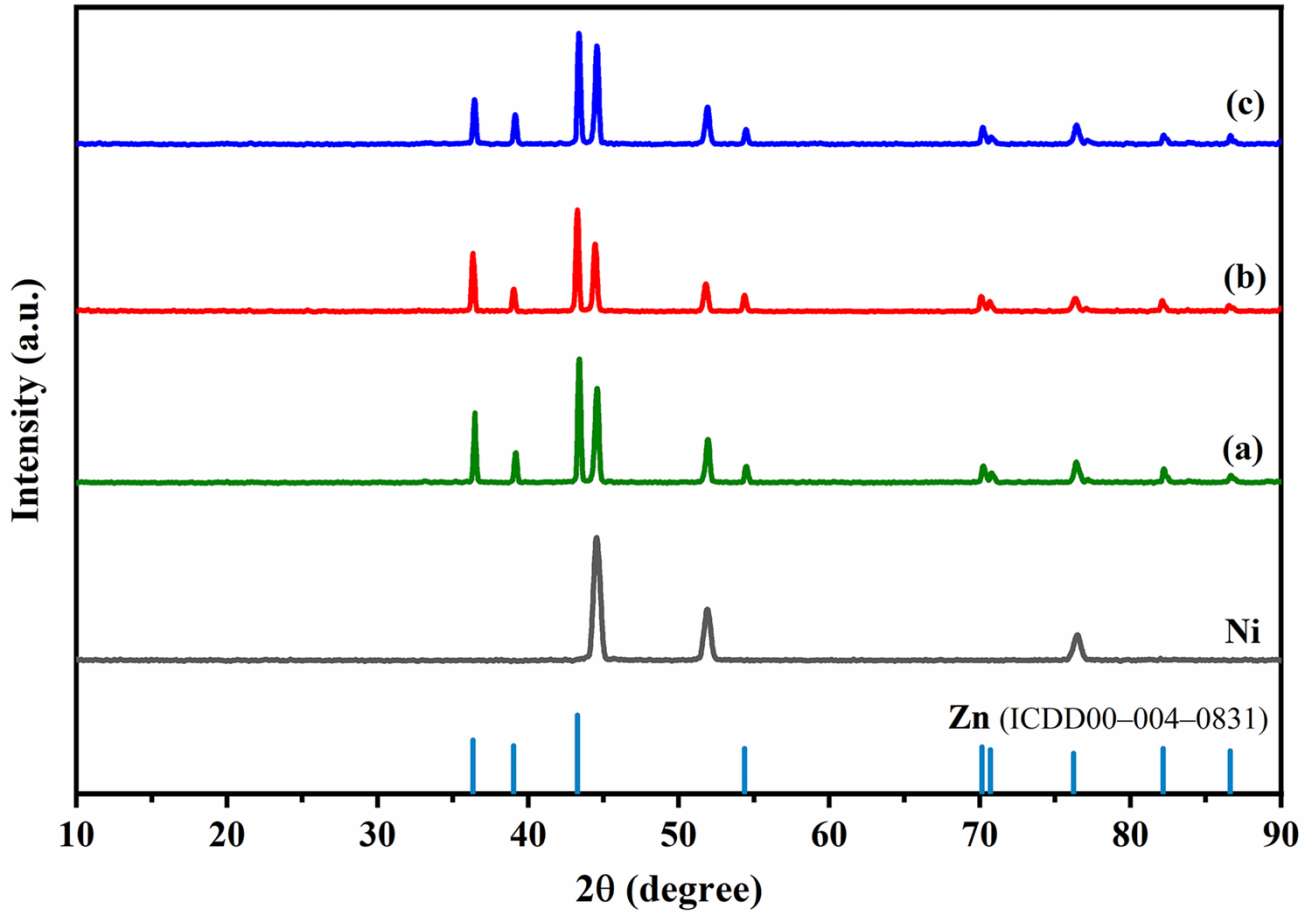
X-ray Diffraction (XRD) patterns (**a**) Zn (**b**) Zn-PPy, and (**c**) Zn-PPy/rGO005.

The crystalline structure of the coated samples is characterized by X-ray diffraction. Figure 2 represents the XRD patterns of the deposited Zn, Zn-PPy and Zn-PPy/rGO005 onto Ni foam substrate. All samples strongly exhibit the diffraction peaks of Ni foam substrate at 2θ of 45°, 52.5° and 76.4°, corresponding to the crystal planes of (111), (200) and (220) in Ni, respectively^41^. Manifestly, the signal intensity of Ni foam substrate reveals the lower in peak intensity after electrodeposition process, attributing to the forming of the deposited materials as shield layer, resulting in lowering Ni signal. For the deposited Zn, the obvious peaks at 2θ of 36.3°, 39.3°, 43.47 and 54.58° are indexed with the crystal planes of (002), (100), (101) and (102), respectively (ICDD00-004-0831)^42^. The diffraction peaks for Zn-PPy and Zn-PPy/rGO005 reveal the similar pattern to that of the deposited Zn but lower in signal intensities of Ni and Zn, suggesting the existence of the deposited PPy and PPy/rGO layer onto zinc surface. Furthermore, Zn-PPy/rGO005 also reveals the increase in the intensity signal at 2θ of 45°, which is a good indexed with (001) reflection of graphitic carbon phase^40^. However, no presence of the diffraction characteristic of PPy at 20° is observed. Similar results are observed for the very low content of the deposited PPy on substrate^43^.

**Figure 3:**
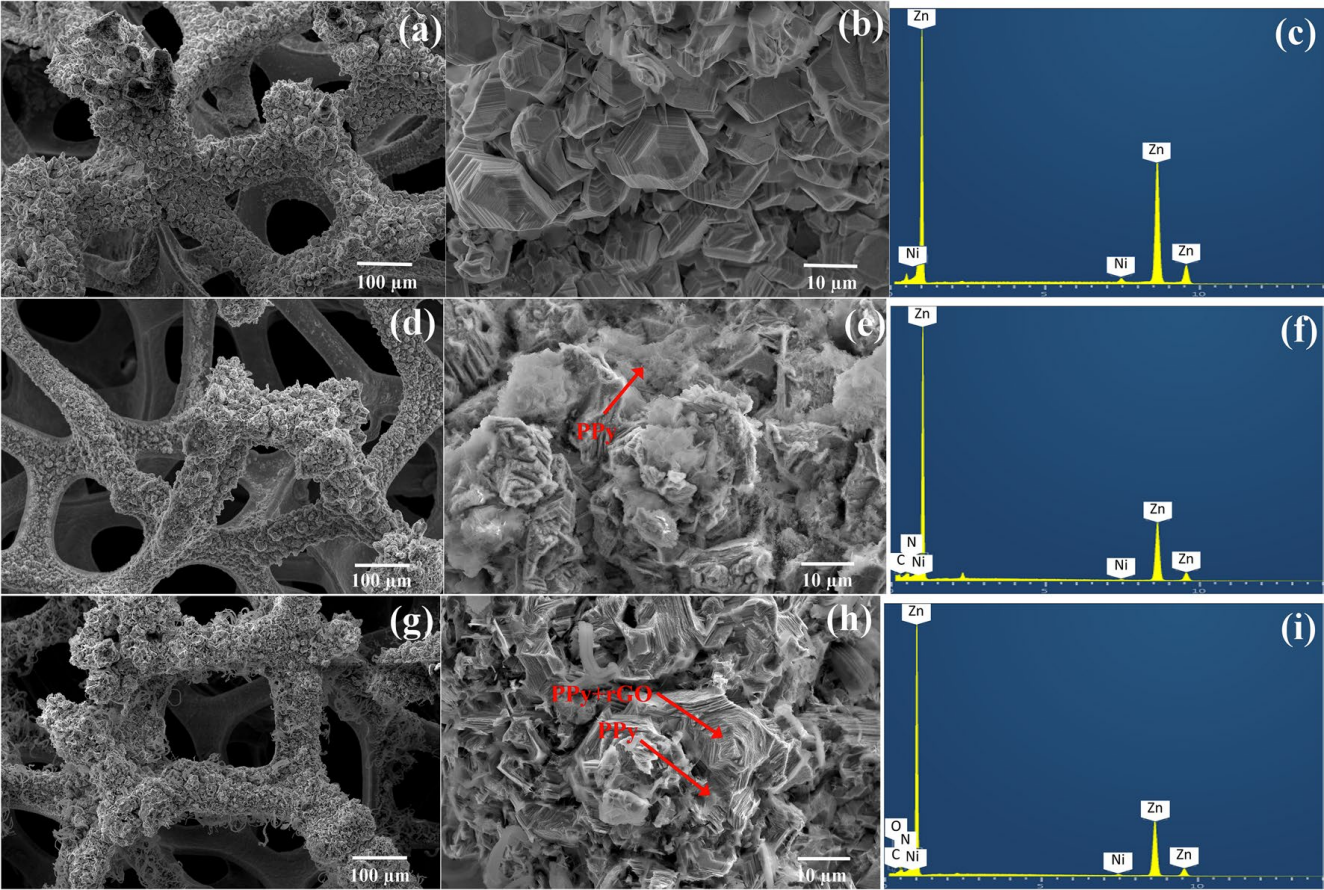
Low, high magnification FESEM images and corresponding element mappings of (**a**–**c**) Zn, (**d**–**f**) Zn-PPy, and (**g**–**i**) Zn-PPy/rGO005.

The field emission scanning electron microscopy/ energy dispersive x-ray spectrometer (FESEM/EDS) images in Fig. 3 demonstrate the surface morphology and element composition of the deposited Zn, Zn-PPy and Zn-PPy/rGO005 onto Ni foam substrate. Principally, Ni foam substrate offers the cross-linked 3D network structure with a high porosity and a high specific surface area for the deposited materials. Figure 3a reveals the numerous deposited zinc grains which are constructed on the Ni porous surface. Obviously, the epitaxial growth of zinc grains consists of the stacked hexagonal plates with the reduced dimension (8–10 µm in size) (Fig. 3b). The element mapping spectrum in Fig. 3c evidently confirms the presence of Zn elements on Ni substrate. For the deposited Zn-PPy, FESEM images in Fig. 3d,e demonstrate the rough flat surface of the coated PPy covering on zinc grain surface. In contrast, the co-deposited PPy/rGO on zinc surface shows the homogenous flat surface of composites coating, indicating the improvement of the coarse PPy propagation on Zn surface in presence of GO (Fig. 3g,h). This result indicates that the high conductivity surface of rGO provides the well-distribution of the co-deposited PPy/rGO on rGO and Zn surface. The surface element analysis of the deposited Zn-PPy and Zn-PPy/rGO005 is further presented in Fig. 3f,i, respectively, evidently confirming the presence of N/C elements for PPy and C/O elements for rGO. In addition, the element color mapping of C and N elements for the deposited Zn-PPy/rGO005 (Fig. S1(c)) reveals the uniform spatial distribution without the presence of cluster and cavity, compared with the deposited Zn-PPy (Fig. S1(b)), suggesting the high effectiveness of the uniform surface for corrosive and dendrite protection^44^. Moreover, these results are consistent with the weight ratio of N element in Fig. S2. The weight ratio of nitrogen atom in Zn-PPy/rGO005 is lower than that of Zn-PPy, attributing to the covering of rGO on PPy of Zn-PPy/rGO composites.

**Figure 4:**
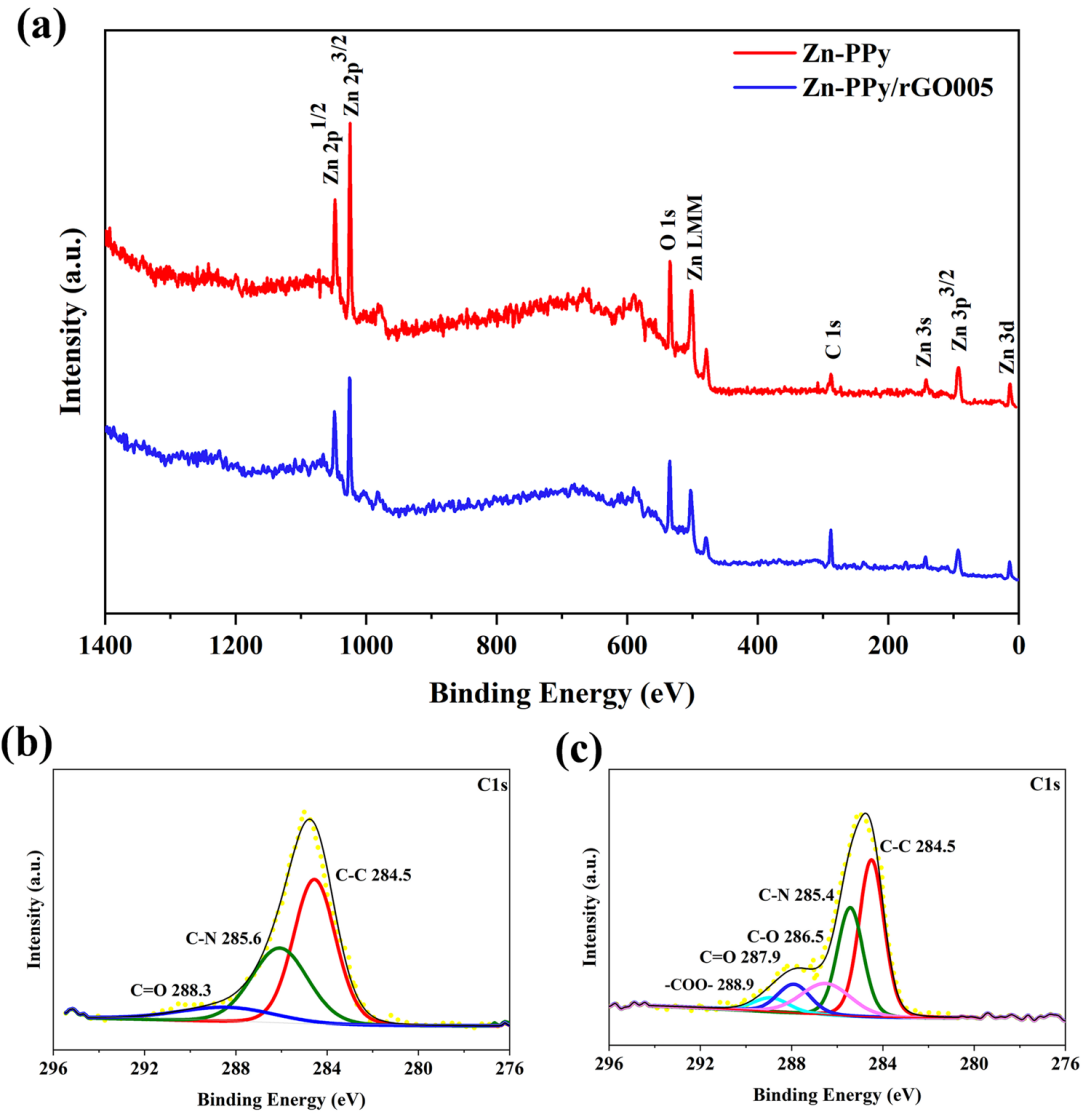
(**a**) XPS survey spectrum of samples (**b**) C1s spectra of Zn-PPy, and (**c**) C1s spectra of Zn-PPy/rGO005.

XPS is used to characterize the chemical state of the as-prepared Zn-PPy and Zn-PPy/rGO005 samples. The XPS survey spectra of the as-prepared samples are displayed in Fig. 4a, obviously confirming the binding energies associated with C1s, O1s, Zn LMM, Zn2p, Zn3p and Zn3d for the as-prepared Zn-PPy and Zn-PPy/rGO005. These results suggest the presence of carbon and Zn element for both samples. However, no existence of the N1s characteristic signal appears, indicating the very thin layer of the coated PPy on Zn as aforementioned before in XRD results. Thus, the binding energies characteristic of carbon is an essentially used to identify PPy and PPy/rGO. As seen in Fig. 4b, the C1s spectrum of Zn-PPy can be assigned into three peaks at 284.5, 285.6 and 288.3 eV, corresponding with C–C, C–N and C=O bond of PPy, respectively^45^. While C1s spectrum of Zn-PPy/rGO005 in Fig. 4c reveals the predominant peak with shoulder, which can be fitted into C–C, C–N, C–O, C=O, and –COO– at 288.9 eV at 284.5, 286.5, 286.5, 287.9 eV, and 288.9 eV, respectively. These noticeable C1s spectrum results are a good agreement with the previous reports of PPy and PPy/rGO materials^46^.

**Figure 5:**
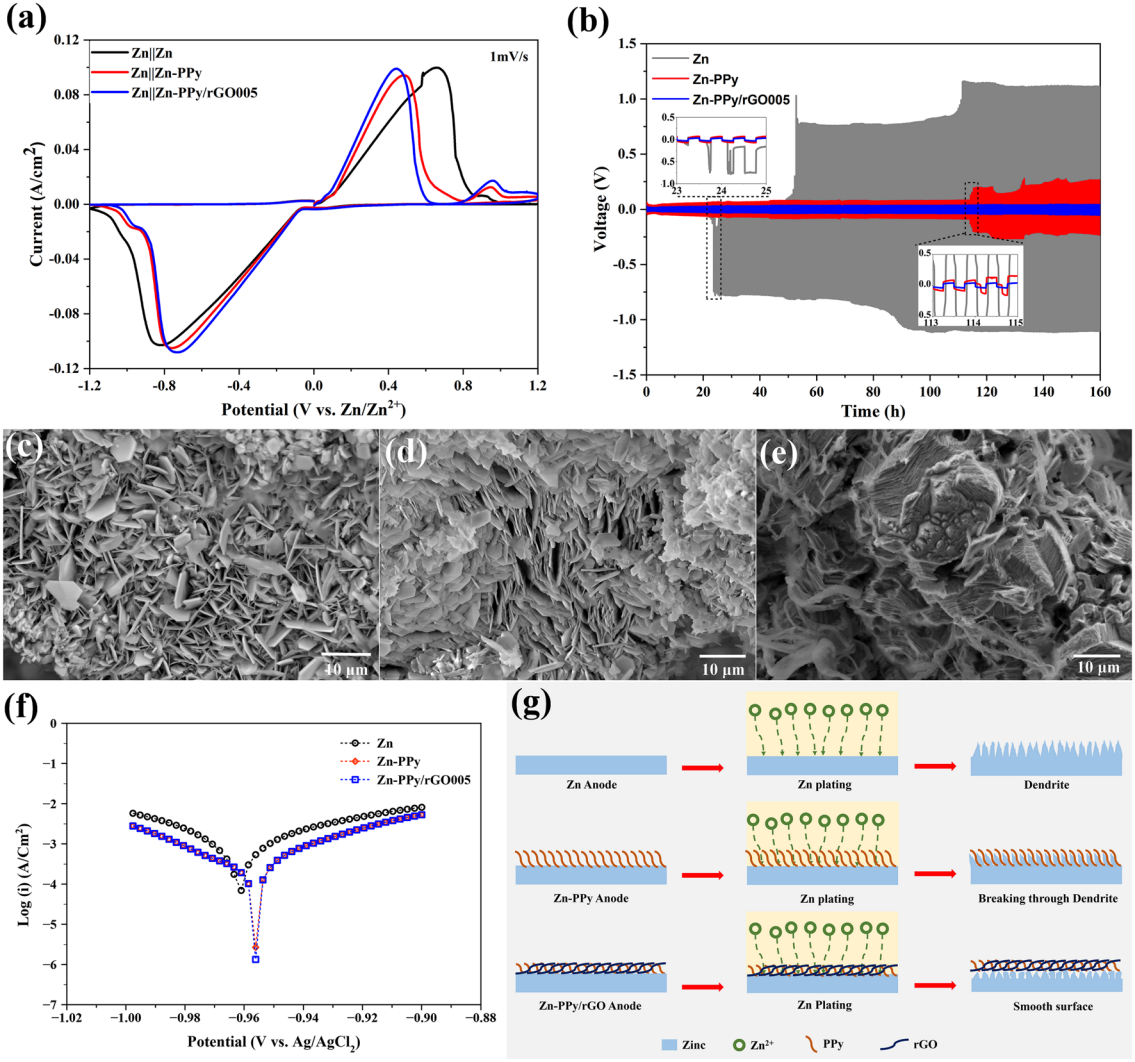
(**a**) Cyclic voltammograms of sample anodes at scan rate of 1.0 mV/s, (**b**) Voltage–time profile of plating/striping process using symmetric cell at current density of 0.5 mA/cm^2^ (inset: enlarged potential profiles at different times), FESEM images of (**c**) Zn, (**d**) Zn-PPy, and (**e**) Zn-PPy/rGO005 anodes after 50 cycles (25 h) of plating/striping process, (**f**) Linear polarization curves of anode samples, and (**g**) Schematic illustrations of the zinc deposition process on anodes.

To investigate the electrochemical behaviors of the as-prepared samples during deposition process, cyclic voltammograms are performed using the symmetrical cell of Zn, Zn-PPy and Zn-PPy/rGO005 samples in 2.0 M ZnSO_4_ electrolyte at scan rate of 1.0 mV/s with the voltage range of − 1.2 to 1.2 V vs. Zn^2+^/Zn. As shown in Fig. 5a, for the positive potential region, two distinct peaks are observed at 0.49 and 0.94 V for Zn-PPy and at 0.44 and 0.96 V for Zn-PPy/rGO005, suggesting the oxidation of Zn^2+^ ions and PPy, respectively. For the negative potential region, Zn-PPy exhibits the peak at − 0.76 V with the shoulder at − 0.97 V. Likewise, Zn-PPy/rGO005 reveals the peak at − 0.73 V with shoulder at − 0.94 V. It is seen that CV curves consisting of the predominated peaks with shoulder can be implied the characteristics of Zn^2+^ ions and PPy reduction, respectively^47,48^. For the CV curve of Zn-PPy/rGO005, a lower-shift potential at the oxidation peak position as well as a higher-shift potential at reduction peak position, compared with the Zn and Zn-PPy, indicates an improvement of coulombic efficiency and fast Zn^2+^ ion plating/stripping on zinc surface^49–51^.

The long-term cycling stability of the as-prepared samples in symmetrical cell is implemented with 15 min in each charge/discharge cycle at 0.5 mA/cm^2^, as shown in Fig. 5b. It is worth noting that the voltage–time curves of Zn reveal a high voltage hysteresis of 89 mV at the beginning and sustains for only 25 h (inset of Fig. 5b). This failure with short-life cycles causes by the short circuit, occurring from the formation of Zn dendrites piercing the separator. In contrast, the voltage–time curves of the as-prepared Zn-PPy and Zn-PPy/rGO reveal the reduced voltage hysteresis of 62 mV for 114 h and 25 mV for 160 h, respectively. Manifestly, the as-prepared Zn-PPy/rGO005 remarkably enhances the electrochemical cycling without the voltage–time fluctuation, suggesting an excellent cycling stability and enhancing the interfacial kinetics of Zn^2+^ ions during plating/stripping process^25,28^. In addition, the morphology evolution of the as prepared Zn samples after 50 cycles is investigated by FESEM images as shown in Fig. 5c-e. After 50 cycles, the presence of sheet-like nanostructures, as well as the formation of dendrites, vertically grow on Zn and Zn-PPy surface (Fig. 5c,d). In contrast, PPy/rGO coated Zn clearly shows dendrite-free and smooth Zn deposition surface as seen in Fig. 5e. The horizontal orientation of rGO benefits the uniform distribution of electrical field for Zn plating/stripping process. These results attribute to the deposited layer of Zn-PPy/rGO005 serving as the protective layer to reduce Zn^2+^ nucleation overpotential, benefitting to suppress dendrite growth and reduce polarization voltage. Furthermore, the cycling performance of the Zn-PPy/rGO005 is comparable to and better than that of the previous reports of the modified Zn anode with MoS_2_^31^.

The Tafel line extrapolation procedure is used to investigate the corrosion properties in which the anode samples is linear polarized in three-electrode system with the open circuit potential range of − 0.1 to − 0.9 V at 0.5 mV/s. The resulted linear polarization curves are shown in Fig. 5f. The corrosion inhibition efficiencies (*η*) of Zn-PPy and Zn-ppy/rGO005 are also calculated by Eq. (1):^52^

1 $$\eta \left(\%\right)=\frac{{I}_{corr}-{I}_{corr}^{^{\prime}}}{{I}_{corr}} \times 100$$

where ${I}_{corr}$ and ${I}_{corr}^{^{\prime}}$ are the corrosion current density of Zn and Zn-PPy or Zn-PPy/rGO005, respectively. As illustrated in Table 1, the corrosion current densities (${I}_{corr}$) of the as-prepared Zn, Zn-PPy and Zn-PPy/rGO005 are 483.2, 155.4, and 149.2 μA/cm^2^. It is obviously seen that Zn-PPy/rGO005 possesses the lowest corrosion current with the highest corrosion inhibition efficiency of 69.1%, indicating superior corrosion resistance. In addition, the linear polarization curves, and the corrosion inhibition efficiencies of Zn-PPy/rGO having the different GO concentrations are presented in Fig. S3 and Table S2, respectively. Comparing with Zn-PPy/rGO005, the corrosion inhibition efficiencies of Zn-PPy/rGO at 0.01 and 0.1 mg/ml GO concentrations demonstrate more corrosion resistance.

**Table 1:** The corrosion inhibition efficiencies (*η*) of anodes.

Anodes	*E*_corr_ (V)	*I*_corr_ (μA/cm^2^)	*η* (%)
Zn	− 0.9617	483.2	–
Zn-PPy	− 0.9562	155.4	67.8
Zn-PPy/rGO005	− 0.9537	149.2	69.1

The mechanism of interface phenomenon for the as-prepared Zn, Zn-PPy, and Zn-PPy/rGO samples is schematically proposed during plating/striping process, as seen in Fig. 5g. For Zn, the growth of dendrite formation is unquestionably on zinc surface during Zn plating process, owing to the uneven flux of Zn^2+^ions leading to the forming of thorn-like surface. As evidently illustrated in Fig. 5b, the unstable overpotential voltages are observed for Zn anode due to excess Zn^2+^ nucleation continually growing on surface. For the deposited PPy on zinc surface, the irregular agglomerated PPy is formed covering on zinc surface as protective layer, causing the breaking through the growth of Zn dendrite on Zn-PPy surface. However, to achieve the long-life cycling stability, the well uniform distribution of the deposited layer is required. Interestingly, this issue can be effectively addressed by covering Zn surface with composite layers of PPy/rGO. Maintaining such high surface area and high conductive surface for homogenized Zn^2+^ ion diffusion during plating/striping process, resulting in the inhibition of dendrite formation. Besides, the 2D graphitic carbon characteristic can act as shield layer, enabling the protection of zinc corrosion from the HER side reaction, which are a good agreement with the corrosion results and previous reports^36,53^.

**Figure 6:**
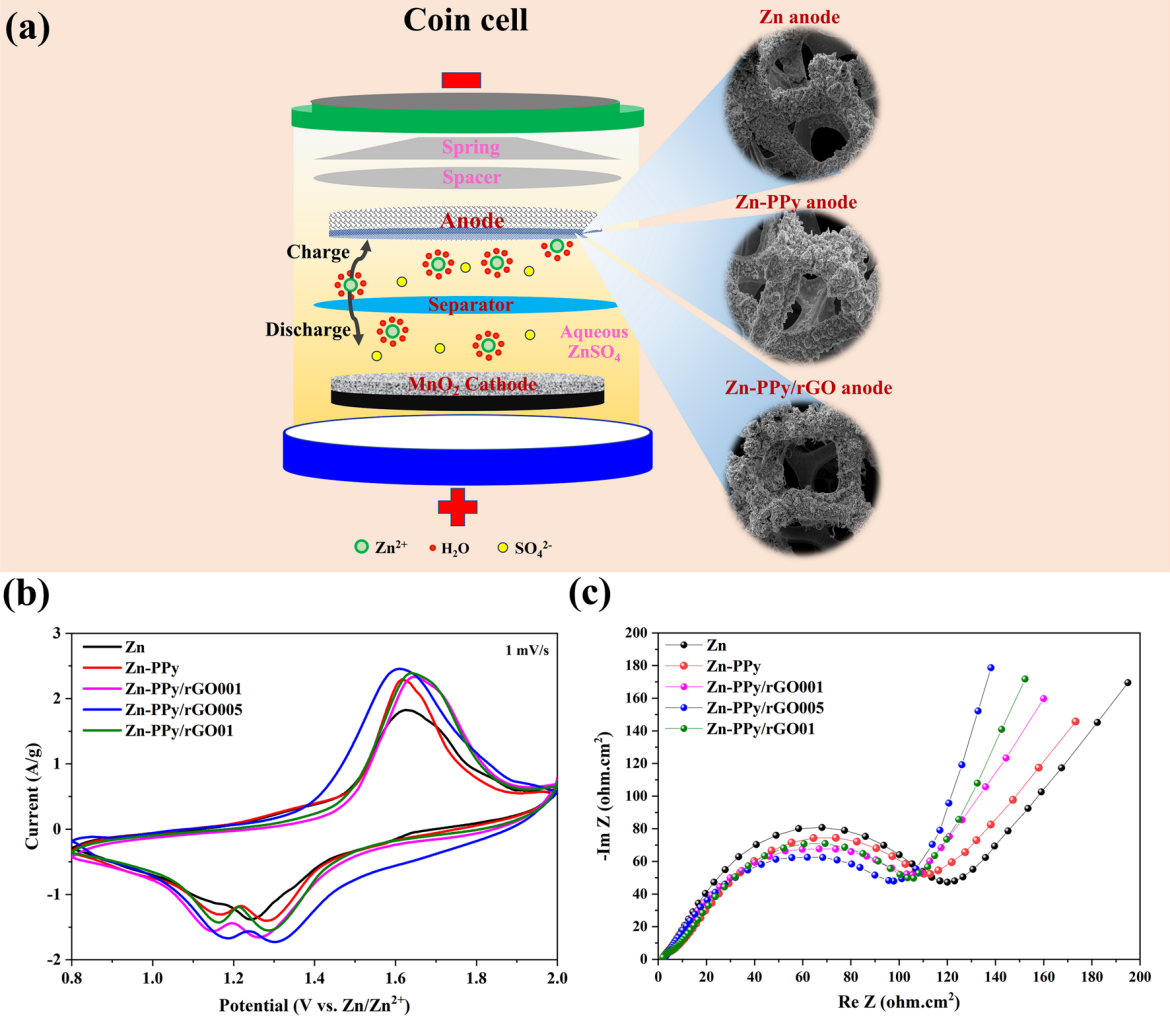
(**a**) Schematics of the chemistry of the zinc-ion battery. The inset on the right shows FESEM images of anodes, (**b**) Cyclic voltammograms at a scan rate of 1 mV/s, and (**c**) Nyquist plot of EIS spectra of coin cell batteries with different anodes.

Figure 6a demonstrates the coin cell batteries in this study, configurating with MnO_2_ as cathode, the deposited Zn and its coated composites as anode, glass fiber as separator and 2.0 M ZnSO_4_ aqueous solution as electrolyte. During discharging, anodic zinc is dissolved in the form of Zn^2+^ ions, rapidly solvated with electrolyte, and diffused through the separator to the MnO_2_ cathode. Then, the solvated Zn^2+^ ions are de-solvated in the form of Zn^2+^ ions and intercalate into MnO_2_ cathode. Similar processes can be reversed when the charging process takes place. Lastly, Zn^2+^ ion deposits back on the zinc anode.

To compare the electrochemical and battery performance of Zn, Zn-PPy, and Zn-PPy/rGO having the different GO concentrations as anode for ZIBs, the different concentrations of GO in electrolyte are varied at 0.001, 0.05 and 0.1 mg/ml (denoted as Zn-PPy/rGO001, Zn-PPy/rGO05 and Zn-PPy/rGO01). Figure 6b shows the CV profiles of the battery with the different corresponding anodes at a scan rate of 1.0 mV/s over the potential range 0.8 to 2.0 V vs. Zn/Zn^2+^. It is seen that two distinct redox peaks are observed during oxidation and reduction reaction, ascribing to the typical characteristics of the electrochemical insertion/extraction of Zn^2+^ and H^+^ ions in MnO_2_ cathode^54,55^. Obviously, the CV curves of Zn-PPy and Zn-PPyrGO anode exhibit a higher peak intensity and a larger enclosed area than that of Zn anode, indicating the better electrochemically active surface area (ECSA). Furthermore, the cathodic and anodic peaks of Zn-PPy/rGO005 are slightly shifted to lower potentials compared with Zn, Zn-PPy, Zn-PPy/rGO01, and Zn-PPy/rGO0001, suggesting the highest conductive surface of the Zn-PPy/rGO005 anode and the fastest kinetic reversibility^56,57^. To emphasize the electrochemical performance, EIS of the coin cell battery with the different anode samples is complemented at open circuit potential over the frequency range 0.05 to 10,000 Hz. Figure 6c displays the typical Nyquist plot of coin cell battery samples, which is divided into three parts: zero-intercept, semicircle-like at a medium to low frequency region, and the incline-straight line at a low frequency region. Principally, the zero-intercept at the real part ascribes to state of battery corresponding to the Zn reaction. The semicircle-like at moderate to low frequency corresponds with charge transfer resistance (R_ct_), arising from the characteristic of Zn^2+^ ions intercalation into the MnO_2_ cathode. Clearly, the electrical conductivity of Zn//MnO_2_ batteries can be improved by coating with PPy and PPy/rGO. The R_ct_ of Zn, Zn-PPy, Zn-PPy/rGO001, Zn-PPy/rGO005, and Zn-PPy/rGO01 anodes are 127, 121, 117, 108 and 116 Ω.cm^2^, respectively. These results suggest the fast kinetics of Zn^2+^ ions of the modified anode corresponding with a plenty of Zn^2+^ ions for insertion^58^. However, the obtained slight increase in R_ct_ value at 0.01 mg/ml rGO is caused by the inhomogeneous surface composites, leading to the inefficient synergistic between rGO and PPy, which are consistent with the SEM, CV, GCD and cycle performance.

**Figure 7:**
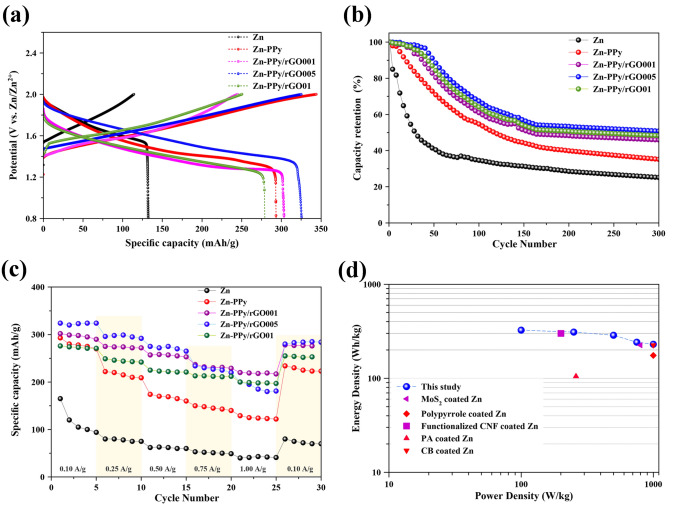
(**a**) Galvanostatic charge–discharge profile at 0.1 A/g, (**b**) Capacity retention of the ZIBs, (**c**) Rate capability at different discharge rates, and (**d**) Ragone plots showing the comparison of the energy densities and power densities of our batteries with the Zn//MnO_2_ batteries reported in the literature using MoS_2_ coated Zn^31^, Polypyrrole coated Zn^22^, Functionalized CNF coated Zn^23^, PA coated Zn^25^ and CB coated Zn^28^ as anodes.

Figure 7a shows the discharge/charge profiles of the sample anodes in coin cell batteries at specific current density of 0.1 A/g over the potential range of 0.8 to 2.0 V. The discharge capacity for Zn-PPy/rGO005 anode is 325 mAh/g, whereas Zn, Zn-PPy, Zn-PPy/rGO001, and Zn-PPy/rGO01 anodes register only 132, 293, 303, and 278 mAh/g, respectively. The slight decrease in capacity at high content of rGO may cause by the presence of the inhomogeneous rough surface of the deposited PPy/rGO01 on Zn surface (see SI, Fig. S4). Thus, all evident results suggest that the ZIBs using Zn-PPy/rGO005 as anode possess the highest energy density, which is in good agreement with the CVs results.

The long-term cycling of the different material coated Zn is tested at 0.1 A/g. The capacity retention is calculated relative to the capacity at initial cycling as shown in Fig. 7b. As expected, the deposited Zn anode exhibits an abrupt capacity fading during the initial 100 cycles and retains only 25% capacity retention after 300 cycles. The deposited Zn-PPy anode reveals the improving of cycling stability with 35% capacity retention after 300 cycles, suggesting the effectiveness of the PPy as protective layer for preventing dendrite agglomeration on Zn surface. Compared with Zn and Zn-PPy anode, the co-deposited PPy/rGO on zinc anodes turn the higher the cycling stability corresponding to the amount of rGO loading, revealing 48, 50 and 46% retention after 300 cycles for Zn-PPy/rGO01, Zn-PPy/rGO05 and Zn-PPy/rGO001, respectively. The slight decrease in cycling stability at high rGO loading can be attributed to the ineffectiveness of inhomogeneous rough surface of PPy/rGO01. Thus, the optimized ratios and the surface characteristic of the deposited PPy/rGO on Zn anode can promote a long-term stability of the ZIBs.

Figure 7c presents the rate performances of ZIBs with the different anodes. The charge–discharge cycling at different specific current densities of 0.1, 0.25, 0.5, 0.75, and 1.0 A/g is employed with five cycles for each rate. Clearly, Zn-PPy/rGO005 anode delivers the highest capacity performance even at high current density. Furthermore, after cycling at high current, when the current turns back to 0.1 A /g, Zn-PPy/rGO005 anode can preserve the almost initial discharge capacity of 280 mAh/g. The rate performances of ZIBs with the different anode samples are in the order of Zn-PPy/rGO005 > Zn-PPy/rGO001 > Zn-PPy/rGO01 > Zn-PPy > Zn. It is clearly that the deposited PPy/rGO on Zn surface is not only to improve the corrosion resistivity of Zn surface but also to enhance in electrochemical performance for ZIBs. Thus, the better rate capability even at high current density of Zn-PPy/rGO anode can be expressed as high surface area and high conductivity features of the deposited PPy/rGO layer, enabling the fast kinetics for Zn^2+^ ion absorption/deposition and improving the well uniform distribution of the deposited Zn during charge/discharge process on composite layer^22,31^.

To further evaluate the performance of various batteries, the Zn//MnO_2_ batteries with different anodes from other previous reports are used to compare with this study. The Ragone plot, which is plotted by the energy density (Wh/kg) versus the power density (W/kg), is shown in Fig. 7d. The results show that the power density of this study reaches the maximum 1000 W/kg with the energy density of 225 Wh/kg, which is superior to the other ZIBs reports. Here, the observed performance in terms of power and energy density can be attributed to the successful tailoring morphology of the composite coating of PPy/rGO on Zn anode, endowing its performance with dendrite suppression feature.

## Conclusion

In conclusion, Zinc based composites anodes were prepared with in-situ electrodeposition of PPy and rGO and their electrochemical performances were investigated in the ZIBs. The formation of the PPy/rGO layer on zinc surface was not only one that protected surface from corrosion but also inhibited the dendrite. The stability test by the plating/stripping for the symmetric cells of Zn-PPy/rGO005 anode revealed more than 160 h at 0.5 mA/cm^2^, which is much better than that of Zn anodes. The kinetic of Zn^2+^ ions reaction and Zn^2+^ ions intercalation was improved by composites coated zinc anode. In addition, ZIBs with Zn-PPy/rGO005 as anode can deliver a prominent discharge capacity of 325 mAh/g, which was superior to Zn anode (132 mAh/g). The capacity retention registered by the Zn-PPy/rGO005 anode was 50% whereas the Zn anode only displayed a capacity retention of 25% after the 300th cycles. Thus, these evident results demonstrate the promising candidate of the conductive composite coating prepared by cyclic voltammetry deposition for advanced Zn anode utilizing for aqueous ZIBs.

## Supplementary Information

Supplementary Information.

## Data Availability

The authors declare that all relevant data are within the paper.
