# Supplementary material for: Polypyrrole/reduced graphene oxide composites coated zinc anode with dendrite suppression feature for boosting performances of zinc ion battery
Source: Sci Rep. 2022 May 23;12:8689. doi: 10.1038/s41598-022-12657-9 (PMC9127107; doi:10.1038/s41598-022-12657-9)
Supplement: Supplementary file 1 — Supplementary Information. [file 41598_2022_12657_MOESM1_ESM.pdf]

Polypyrrole/reduced graphene oxide composites coated zinc anode with dendrite suppression feature for boosting the performances of zinc ion batteries

Sonti Khamsanga[1], Hiroshi Uyama[2], Weerapong Nuanwat[1, 3], Prasit Pattananuwat[1, 3, 4*]

[1] *Department of Materials Science, Faculty of Science, Chulalongkorn University, Bangkok, 10330, Thailand*

[2] *Department of Applied Chemistry, Graduate School of Engineering, Suita, Osaka 565-0871, Japan*

[3] *Center of Excellence on Petrochemical and Materials Technology, Chulalongkorn University, Bangkok, Thailand*

[4] *Research Unit of Advanced Materials for Energy Storage, Chulalongkorn University, Bangkok, Thailand*

Correspondence and requests for materials should be addressed to P. Pattananuwat (email: Prasit.Pat@chula.ac.th)

Supplementary Information

(a)

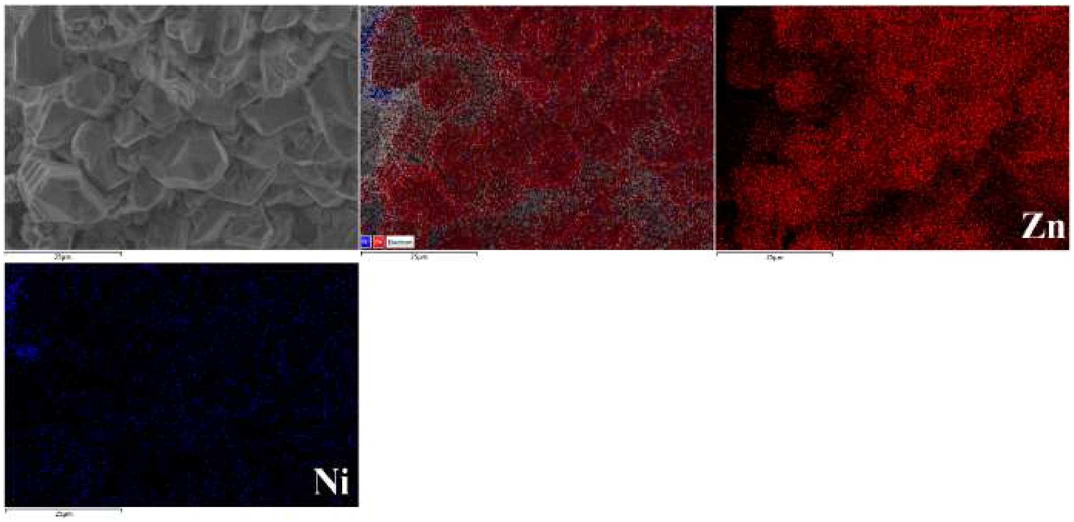

(b)

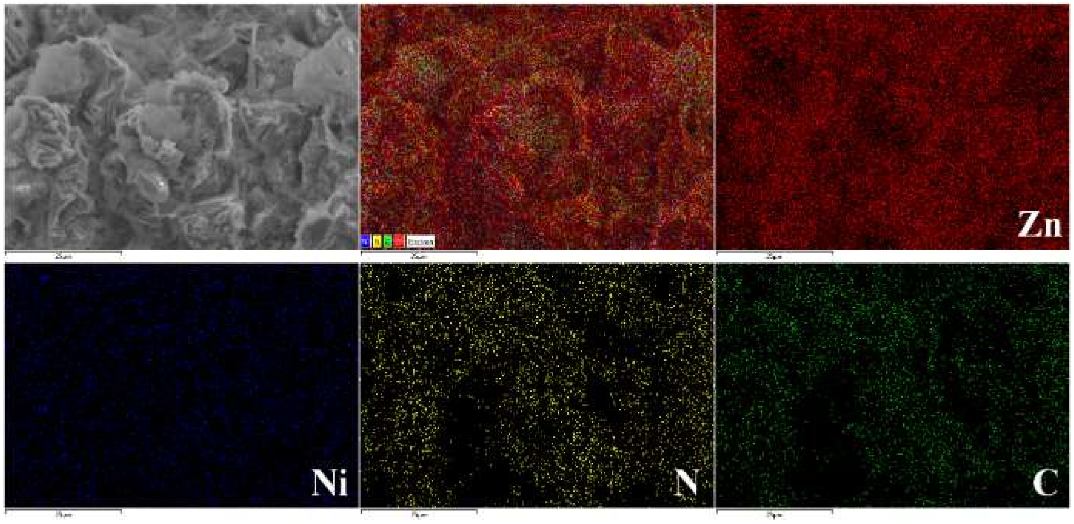

(c)

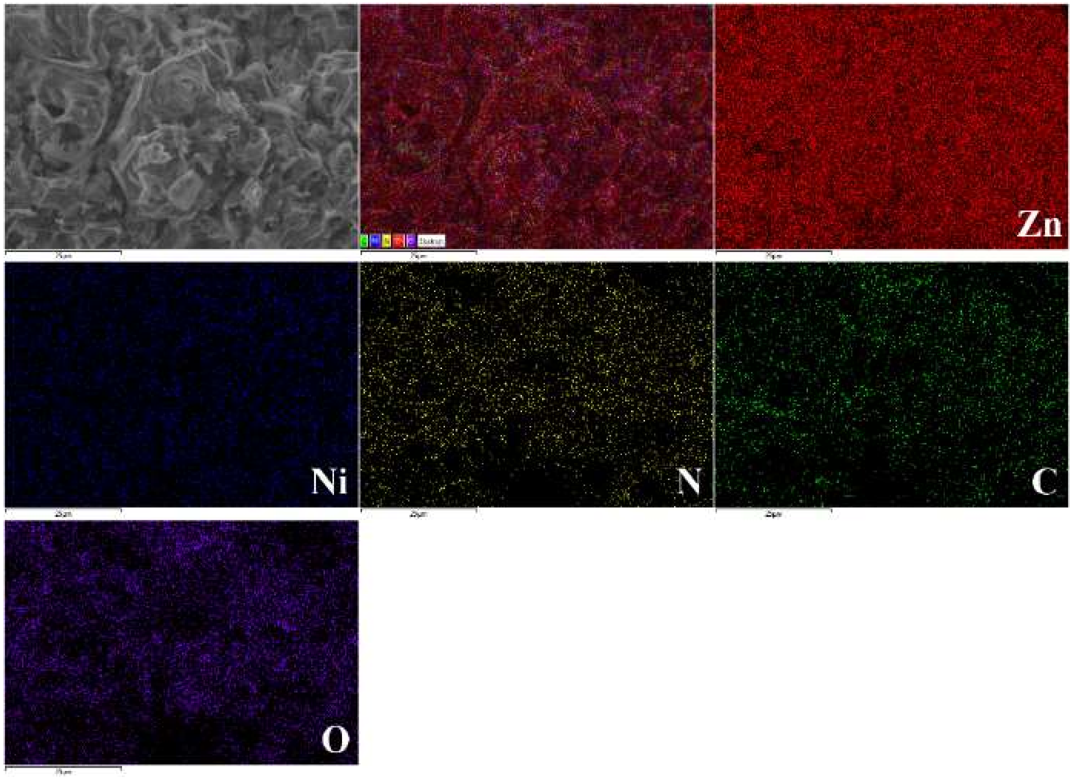

Figure S1. EDS-mapping images of (a) Zn (b) Zn/PPy and (c) Zn/PPy-rGO005.

(a)

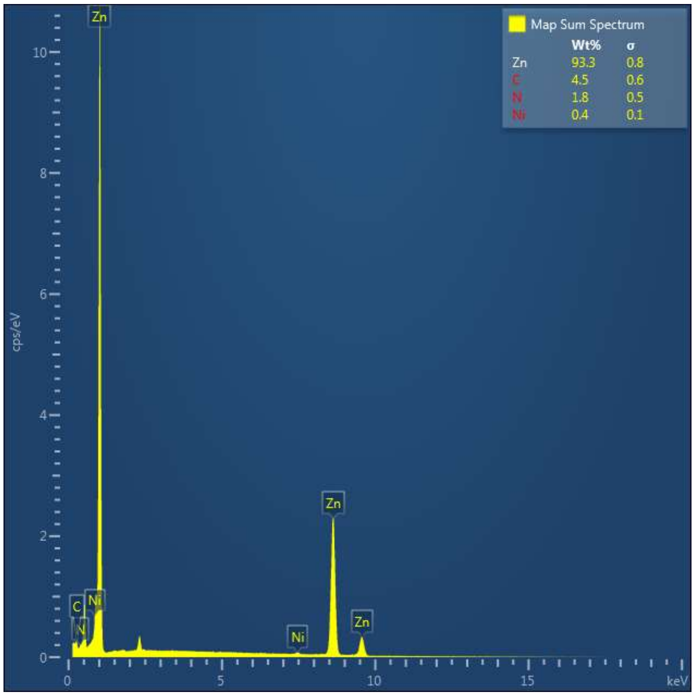

(b)

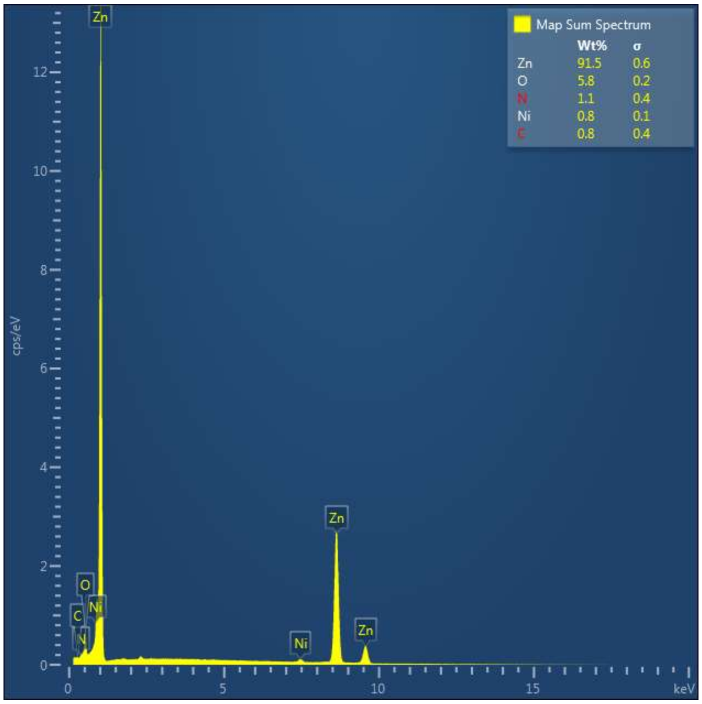

Figure S2. Element-mapping with weight ration of (a) Zn/PPy and (b) Zn/PPy-rGO005

Table S1. The corrosion inhibition efficiencies (η) of Zn-PPy anodes

Anodes	E_{corr} (V)	I_{corr}(μA/cm^2)	η (%)
**Zn**	-0.9617	483.2	-
**Zn-PPy3**	-0.9601	160.1	66.8
**Zn-PPy5**	-0.9562	155.4	67.8
**Zn-PPy7**	-0.9582	179.8	62.7

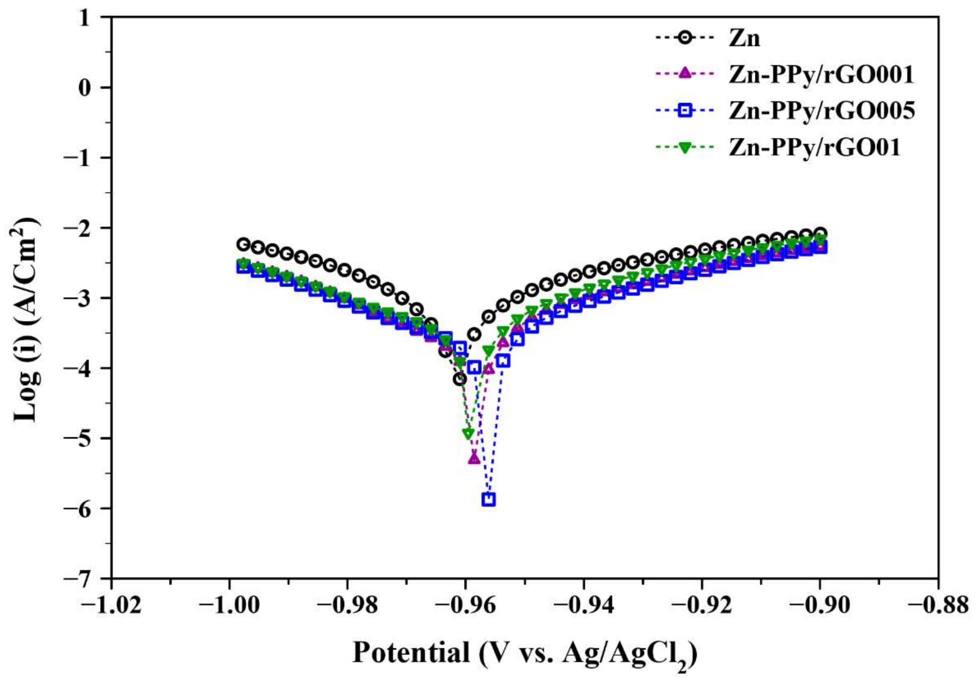

Figure S3. Polarization curves of Zn-PPy/rGO anodes by various concentration of rGO: Zn-PPy/rGO001-0.01 mg/ml, Zn-PPy/rGO005-0.05 mg/ml and Zn-PPy/rGO01-0.1 mg/ml.

Table S2. The corrosion inhibition efficiencies (η) of Zn-PPy/rGO anode.

Anodes	E_{corr} (V)	I_{corr}(μA/cm^2)	η (%)
**Zn**	-0.9617	483.2	-
**Zn-PPy/rGO001**	-0.9585	153.4	68.2
**Zn-PPy/rGO005**	-09537	149.2	69.1
**Zn-PPy/rGO01**	-0.9596	154.8	67.95

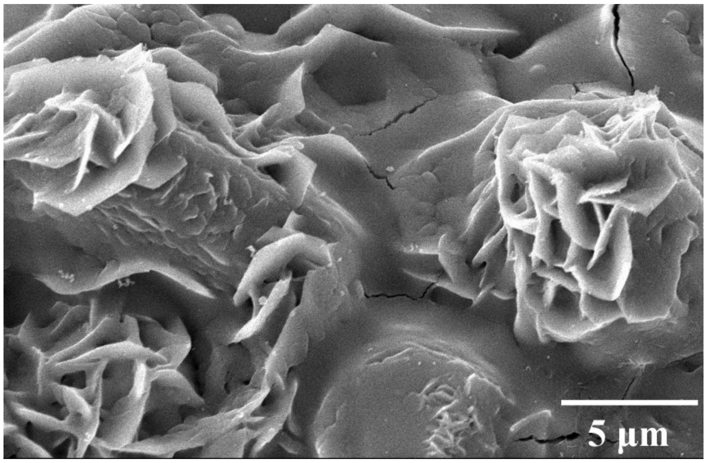

Figure S4. SEM images of Zn-PPy/rGO01.
